# Wild Edible Fungi in the Catalan Linguistic Area: A Scoping Review Linking Nutritional Value to Ethnomycology

**DOI:** 10.3390/foods14162897

**Published:** 2025-08-20

**Authors:** Canòlich Álvarez-Puig, Joan Casamartina, Teresa Garnatje, Manel Niell, Airy Gras, Joan Vallès

**Affiliations:** 1 Laboratori de Botànica–Unitat Associada CSIC, Facultat de Farmàcia i Ciències de l’Alimentació–Institut de Recerca de la Biodiversitat (IRBio), Universitat de Barcelona, 08028 Barcelona, Catalonia, Spain; 2 Institut Botànic de Barcelona (CSIC-CMCNB), 08038 Barcelona, Catalonia, Spain; 3 Jardí Botànic Marimurtra-Fundació Carl Faust, 17300 Blanes, Catalonia, Spain; 4 Andorra Recerca i Innovació (AR+I), AD600 Sant Julià de Lòria, Andorra; 5 Secció de Ciències Biològiques, Institut d’Estudis Catalans, 08001 Barcelona, Catalonia, Spain

**Keywords:** Catalan linguistic area, ethnomycology, nutritional values, traditional food, wild edible fungi, wild food

## Abstract

The Catalan Linguistic Area (CLA) is a mycophile region where interest in the nutritional properties of traditional edible fungi is steadily growing, driven by their gastronomic appeal. The present study undertakes a scoping review with two main objectives. First, to compile a list of edible fungi taxa identified in the CLA, and second, to determine whether their nutritional values have already been published. Data were collected through books from different library catalogues and archives, expert consultations, a specialized database, and a search in three academic databases: PubMed, Scopus and Web of Science. As a result, we obtained a list of 643 culinary fungi taxa, of which 35.46% have reported nutritional values. Moreover, among the most cited CLA culinary fungi, *Hygrophorus latitabundus* Britzelm. and *Hypomyces lateritius* (Fr.) Tul. & C. Tul. have no nutritional values reported in the literature. Additionally, an ethnomycoticity index (EMI) and ethnomyconymic diversity index are proposed as adaptations to ethnomycology of two commonly used ethnobotanical indices. To conclude, wild edible fungi (WEF) are widely used in the CLA, but nutritional values for the majority of macromycetes are still lacking. Further studies need to be carried out regarding ethnomycology, enhancing their nutritional values, since data recorded are disperse and difficult to standardise.

## 1. Introduction

Wild edible fungi (WEF) have been collected and consumed as a delicacy by people for thousands of years, probably for their taste and pleasing flavour [1,2]. In colloquial speech, the terms “mushroom” and “fungi” are often used interchangeably, but they have different meanings [3]. Mushrooms can be defined as a kind of macrofungi or macromycetes, characterized by hypogeous or epigeous fruiting bodies, which are part of a larger organism, the fungus, composed of hyphae [3,4]. Concretely, macromycetes are considered to be the group of fungi that develop a fruiting body, formed above or below ground, larger than one millimetre in size and with visibly spore-bearing structures [5,6]. This group includes mainly ascomycetes and basidiomycetes with some information regarding toxicity, edibility, medicinal properties or other ecological functions [5].

As Magbekem et al. [7] pointed out, “all mushrooms are fungi, but not all fungi are mushrooms”. Regarding the diversity of fungi, Hawksworth and Lücking [8] estimated the occurrence of approximately 2.2 to 3.8 million fungi worldwide, with 120,000 accepted fungal species. Mueller et al. [6] estimated a total of 56,679 macrofungi worldwide, and more than half (61.75%) are estimated to be unknown. After providing this overview, in the present work we will use the term WEF to refer to all types of fruiting bodies (ascocarps and basidiocarps) considered edible in the studied area, and the term “macromycete” to refer to all fungi with large (macroscopic) sporocarps [9], popularly known as mushrooms, “*bolet*” in the Catalan language.

Although some communities have a long tradition of using WEF as food, medicine, and source of income [10], it was not until 1957 that Wasson and Wasson [11] formally distinguished ethnomycology as a separate field from other ethnobiological sciences. Nowadays, ethnomycology is described as the scientific discipline that studies traditional knowledge and uses of fungi from a historical and sociological perspective [12,13]. Although fungi are genetically closer to animals than to plants [3], they are often studied alongside plants in ethnobotanical prospections [14]. While Illana [13] considered ethnomycology a subfield of ethnobotany, or a part of it, most authors regard it as a branch of ethnobiology [12,15,16,17], as ethnomycological studies have documented the traditional use of fungal species across cultures for millennia, as in medicine, nutrition, spirituality, and daily life. For example: the uses of *Ganoderma lucidum* (Curtis) P. Karst. (*reishi/lingzhi*) to enhance immune function, increase vitality, and combat fatigue in Chinese medicine [18]; the use of *Claviceps purpurea* (Fr.) Tul. sclerotia in obstetrics, reported in Andorra [19], a part of our studied area; the uses in spiritual and ritualistic uses of psilocybin-containing mushrooms used in Latin America or *Amanita muscaria* (L.) Lam., employed in Siberian shamanic rituals; or the use of *Fomes fomentarius* (L.) Fr. as tinder [20,21].

Leaving aside medicinal or other applications, Kim and Song [22] highlight the increasing global interest in the use of WEF. However, our current understanding of them is still limited—particularly when compared to that of animals and plants—mainly due to a relative scarcity of dedicated research [15,22].

At least 3000 fungal species have gastronomic uses [2] and a conservative estimate shows that 1068 macromycetes species are being used for food alone [3]. Wild edible fungi (WEF) have rich nutritional value with high content of proteins, vitamins, mineral, fibres, low/no calories and cholesterol, and some essential mineral nutrients which are considered as key factors for the normal functioning of the body [2,4]. Nowadays, the use of fungi in our culinary dishes is gaining importance in mycophile areas, and it is also a way to enhance cultural and popular heritage in our cuisine. However, it remains unclear what information the literature provides about their nutritional values in a focused context.

In Europe, the Catalan linguistic area (CLA) is one of the best-studied places when it comes to ethnobotany [23]. Due to the narrow relation between ethnobotany and ethnomycology, as mentioned, we consider that this territory may contain extensive WEF information. Wasson and Wasson [11] classified cultural groups or societies based on their attitudes towards mushrooms, distinguishing them between mycophiles and mycophobes. Mycophile societies exhibit a strong interest in macromycetes, engaging in their collection, preparation, and consumption, whereas mycophobe societies tend to reject them, out of fear and dislike [3]. Niveiro et al. [24] noted that Europeans have centuries of experience in the collection and consumption of fungi. Accordingly, the CLA, and specially Catalonia, is considered a mycophile area [12,25,26], as it recognizes and consumes a wide variety of fungal species.

For this reason, a scoping review was first conducted to compile all culinary macromycetes reported in the CLA—with an only one exception for *Hypomyces lateritius* (Fr.) Tul. & C. Tul., a parasite that grows on the gills of *Lactarius* species. Although it is clearly a micromycete, traditional knowledge considers it a variant of *Lactarius*, rather than a distinct species. Therefore, we included it in the study group. Subsequently, all available nutritional values concerning WEF in the study area were systematically reported.

The following research question was formulated: what are the WEF from the CLA, and what is known from the literature about their nutritional composition?

To answer the question presented, two main objectives were proposed:

1. Elaborate a corpus of the WEF from the CLA;
2. Conduct a scoping review to determine which WEF from the CLA region have reported nutritional values.

## 2. Materials and Methods

### 2.1. Study Area

**Figure 1:**
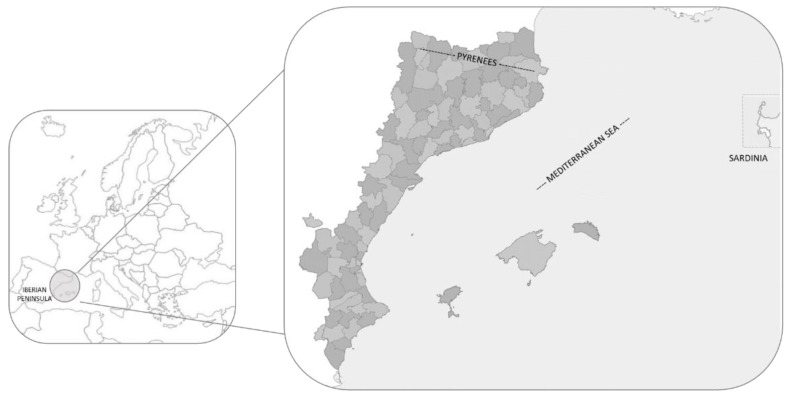
Map of the CLA in the European context.

The Catalan linguistic area (CLA), also known as the Catalan-speaking area, Catalan-language territories, or Catalan Countries, is located in the eastern section of the Iberian Peninsula, the Balearic Islands, a portion of the northern Pyrenees and the city of Alghero (Sardinia); Figure 1 [27]. These territories are spread across four countries: all of Andorra; part of south-east France (Northern Catalonia or the Eastern Pyrenees department); a city in Italy (Alghero in Sardinia); and several regions in Spain, including the Balearic Islands, Carxe (a small area in Murcia), Catalonia, a portion of eastern Aragon, and Valencia [28].

These territories have an extension of around 70,000 km^2^ [29] and around 15,000,000 inhabitants in 2024 [30,31,32,33]. Diversity in the territory spans from sea level along the Mediterranean Sea up to 3143 m a.s.l. at Pica d’Estats in the Pyrenees. According to Llimona [34], approximately 4200 macromycetes’ taxa are present in the study area. These are the taxa included in the Ascomycota and Basidiomycota divisions (the so-called “*bolets*” in the studied territory), some of which are used as food.

### 2.2. Study Design and Data Source

A protocol for this scoping review was developed to guide the research process, including research questions, search strategy, eligibility criteria, and data extraction methods. To ensure transparency and rigor, we followed the PRISMA-ScR guidelines [35] and documented all stages of the review process, including the search strategy, study selection, and data extraction methods. Any deviations from the original plan were noted and justified during the review process. For further details, the protocol can be requested from the corresponding author.

To identify potentially relevant documents, the following bibliographic databases were searched until 3 May 2025: PubMed, Scopus, and Web of Science. The search strategies were drafted and refined through team discussion and with the support of *Centre de Recursos per a l’Aprenentatge i la Investigació de la Universitat de Barcelona* (CRAI UB) services from the University of Barcelona (UB) [36]. The final search strategies for both parts can be found in the Section 2.3. The final search results were exported into Zotero 6.0.36, and duplicates were removed using the program tools and revised by the main author. Moreover, to complete the first search, we also collected information from a specialized working database, internal to our research team, with some data published in https://etnobotanica.iec.cat (accessed on 29 June 2025) [37], and from two library catalogues and archives, *Cercabib* from *CRAI UB* [38] and *Explora la BC* from *Biblioteca de Catalunya (BC)* [39].

### 2.3. Search Strategy

In the first part, the final search for the three academic databases (PubMed, Web of Science and Scopus), was the following: ((fungi OR mushroom) AND (edible OR food OR culinary) AND (Alghero OR Alguer OR Andorra OR Arago* OR “Balearic Islands” OR “Illes Balears” OR Majorca OR Mallorca OR Minorca OR Menorca OR “Pityusic Islands” OR Pitiüses OR Ibiza OR Eivissa OR Formentera OR Carche OR Carxe OR Murcia OR “Catalan Countr*” OR “Països Catalans” OR Catalonia OR Catalunya OR “North* Catalonia” OR “French Catalonia” OR “Catalunya del Nord” OR “Eastern Pyrenees” OR “Pirineus Orientals” OR “Valencia* Community” OR “País Valencià”)) NOT (medic*). The search was slightly adapted for each database. As an example, MeSH (*Medical Subject Headings*) terms from PubMed were included in this database search, to obtain more accurate results.

For the second part of the work, the final search run in the same three databases was the following: ((fungi OR mushroom) AND (“nutri* value” OR “nutri* composition”) AND (edible OR food OR culinary)) NOT (medic*). As mentioned before, the main search was adapted for each database. As another example, in Scopus the search was restricted to the fields TITLE-ABS-KEY, meaning only documents in which the search terms appeared in the title, abstract or author keywords.

### 2.4. Eligibility Criteria

The types of evidence sources included in this review are all those that comprise WEF. In the first search, sources identifying WEF in the CLA were included. The CLA includes Catalonia, Valencia, the Balearic Islands, Andorra, the Roussillon, and Alghero. Peer-reviewed articles, books, ethnobotanical databases, and library databases that mention that a macromycete is edible were included as a bibliographic report (BR). If a taxonomic name was not provided, the fungus was still included, based on its vernacular name, as correlations could be made using other sources or consulting experts. No restrictions were placed on language or publication date. Sources that did not explicitly mention edibility (e.g., those focusing solely on toxicity, ecology, or other non-edibility-related aspects) or sources outside the geographic scope of the CLA were excluded.

In the second search, among the edible fungi identified previously, those with published nutritional values (macronutrients and/or micronutrients) were detailed, with their nutritional information. The data accepted in this part of the work includes nutritional values from work carried out outside the CLA (worldwide). Peer-reviewed articles were included.

In both searches, sources were excluded if they were duplicates, review articles, or lacked sufficient information to determine either edibility or nutritional values. In the second search, some articles were excluded due to the inability to standardize the reported nutrient data.

### 2.5. Study Selection and Data Extraction

Articles were screened and selected by the main author, to minimize different interpretations. To ensure the correct classification of the articles, Zotero collections were used, and articles were classified as included or excluded. Excluded articles were assigned a “reason for exclusion”.

A data-charting form was developed, to determine which variables were extracted. In the first search, for each taxon, we registered the scientific name, and all the vernacular names reported. All the data were organized in an Excel spreadsheet and structured in four columns: taxa as source, vernacular name, reference, and complete reference. To complete these data, it was essential to standardize some of the taxa into a single, to remove duplicate taxa due to old taxonomic systems, for example. As a result, a fifth column was added, with the standardized taxa. This process was carried out mainly with the Index Fungorum [40] database, and Mycobank [41] database as a second option. With these databases, we identified the current names from each taxon, updating all the CLA fungi taxa. All collected data can be consulted in CORA (the Catalan Open Research Area) (see Data Availability Statement Section).

A second data-charting was developed to classify the different nutritional values obtained in the articles selected. The information included in this data-charting was determined following the FAO/INFOODS Food Composition Database for Biodiversity—Version 4.0 (BioFoodComp4.0) [42]. As a result, we collected information about taxa, country and location where the fungi were collected, season, part of the fungi (fruiting body, cap or stipe), processing (raw, dried, processed or frozen), food components, reference and complete reference.

Food component is a term to refer to nutritive and non-nutritive components present in foods [43]. Based on this classification, we classified the nutritional values in the following: energy (kJ and kcal), macronutrients (water, protein, fat, carbohydrates), fibre (total, insoluble, soluble), ash, minerals (Ca, Cu, Fe, K, Mg, Na, Mn, P, Se, Zn), trace elements (Co, Cr), contaminants (As, Cd, Pb), other potentially toxic elements (Al, Ni), and vitamins–thiamine, riboflavin, niacin, vitamin B_6_, vitamin B_12_, ascorbic acid (vitamin C), folate, vitamin A, β-carotene, vitamin D_2_ (ergosterol), vitamin D_3_ (cholecalciferol), and vitamin E (as total tocopherols).

The nutritional values were reported as 100 g on dry matter (DM), since in the scientific literature this is the most used [18]. In some micronutrients, mainly vitamins, data was reported as mg/g DM or µg/g DM. The data were not standardized according to INFOODS tagnames [44]; however, the carting order followed the structure used in BioFoodComp4.0 [42], as specified above. All the nutritional data can be consulted in CORA (see Data Availability Statement Section).

### 2.6. Data Analysis

All descriptive statistics and quantitative ethnobotany were carried out using Excel (Microsoft Excel 365-2025). We calculated the percentage of macromycetes present in the CLA which have a traditional food use, based on the above-mentioned estimation of ca. 4200 taxa existing in this territory (see Section 2.1). We propose the term *ethnomycoticity index* (EMI) as an adaptation of the ethnobotanicity index (EI) [45], originally applied to plants, for use in ethnomycology.

$$EMI=\frac{Number\;of\;macromycetes\;with\;uses\;in\;the\;studied\;area}{Number\;of\;macromycetes\;in\;the\;studied\;area}\times 100$$

In the present paper, we calculated the EMI for food uses in the CLA. Similarly, we calculated what we propose to call the *myconymy diversity index*, adapted to fungi from the phytonymy diversity index [46], and consisting of

$$Myconymy\;diversity\;index=\frac{Number\;of\;vernacular\;names\;with\;uses\;in\;the\;studied\;area}{Number\;of\;macromycetes\;with\;uses\;inthe\;studied\;area}$$

## 3. Results and Discussion

### 3.1. Study Selection

**Figure 2:**
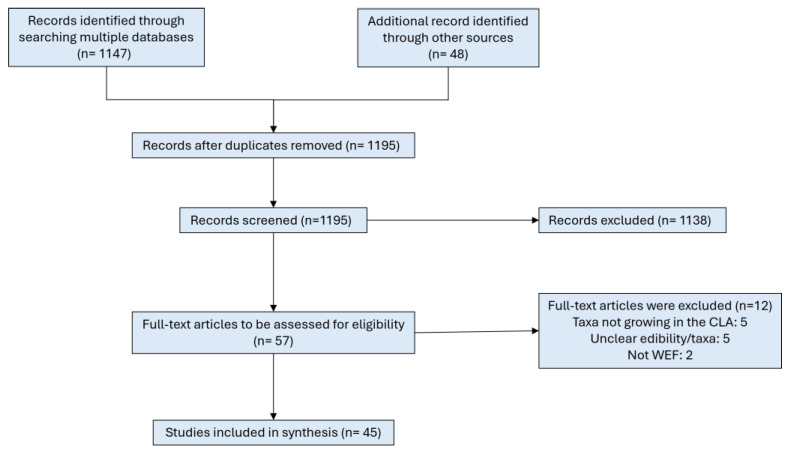
Flowchart of the identification of CLA edible fungi.

Regarding the first search, after duplicates were removed, a total of 1195 references of articles and books were identified from searches of academic and specialized databases, library catalogues and archives, and expert consultation. Based on the title of the references, 1138 were excluded at the initial stage, with 57 full text sources to be retrieved and assessed for eligibility. Of these, twelve were excluded for the following reasons: five reported edible macromycetes not growing in the CLA, five lacked sufficient edibility data or identifiable taxa, and two did not contain WEF. The remaining 45 studies were considered eligible to elaborate a corpus of WEF in the CLA. Figure 2 provides a schematic overview of source selection from the first search.

**Figure 3:**
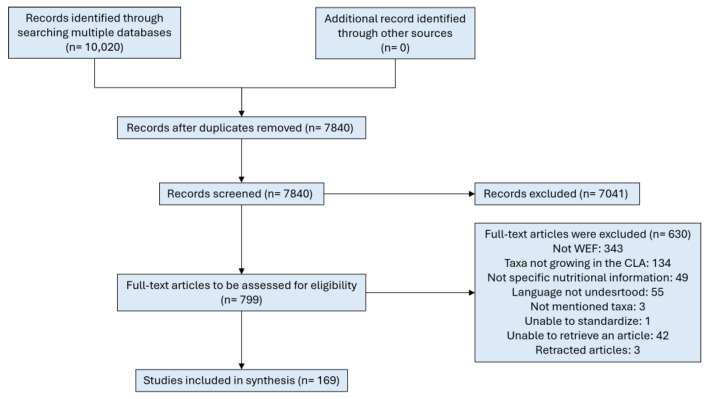
Flowchart of the identification of nutritional values from CLA edible fungi.

Once the first search had been completed and analysed, a second was carried out to find which WEF from the CLA have nutritional values published. After duplicates were removed, a total of 7840 articles were identified from multiple databases. Based on the title of the references, 7041 were excluded at the initial stage, including reviews, with 799 full-text sources to be retrieved and assessed for eligibility. Of these, 630 were excluded for the following reasons: 343 did not contain WEF data, 134 reported edible macromycetes not growing in the CLA, 49 did not indicate nutritional values, 55 were written in languages neither familiar to the authors nor corresponding to their native language, 3 articles were excluded due to our inability to assign taxa, and 1 article did not have sufficient data to standardize the results in our data-charting method. Finally, 42 studies were excluded because we were unable to retrieve them and 3 articles were retracted, resulting in a final selection of 169 articles eligible for this review. Figure 3 provides a schematic overview of source selection from the second search.

As mentioned in Section 2.5, characteristics and results of individual sources of evidence are available in CORA (see Data Availability Statement Section).

### 3.2. WEF Corpus from the CLA

The initial phase of research and data collection has enabled the compilation of a total of 6088 BR in an Excel spreadsheet, corresponding to 910 initial taxa. For the purposes of this study, a BR is defined as each instance in which a mushroom species is mentioned or cited in any of the bibliographic sources consulted. Following the completion of the taxonomic standardization process, a total of 643 taxa—627 at the species level, and 16 at the genus level—have been reported as edible mushrooms of the CLA. Additionally, one section (*Lactarius* sect. *Deliciosi* (Fr.) Redeuilh, Verbeken & Walleyn) was also designated as edible, since it constitutes an ethnotaxon, in the sense that its species are not distinguished by the informants, who call all of them by the same vernacular names. Ethnotaxa are rather frequent in ethnomycology [16,17].

**Figure 4:**
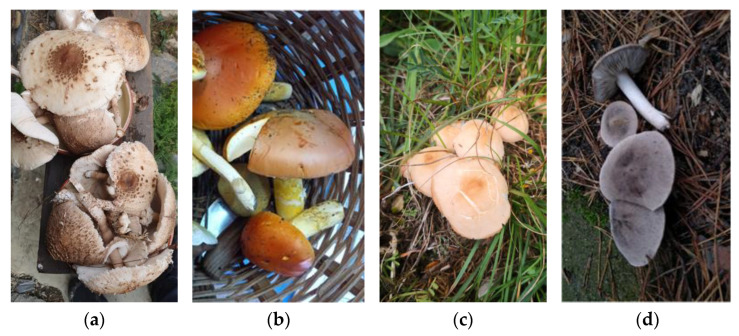
Four of the most cited wild edible fungi (WEF) in the Catalan linguistic area (CLA): (**a**) *Macrolepiota procera* collected in the forest; (**b**) *Amanita caesarea* in a traditional basket; (**c**) *Marasmius oreades* in meadows; (**d**) *Tricholoma terreum* in the forest.

**Figure 5:**
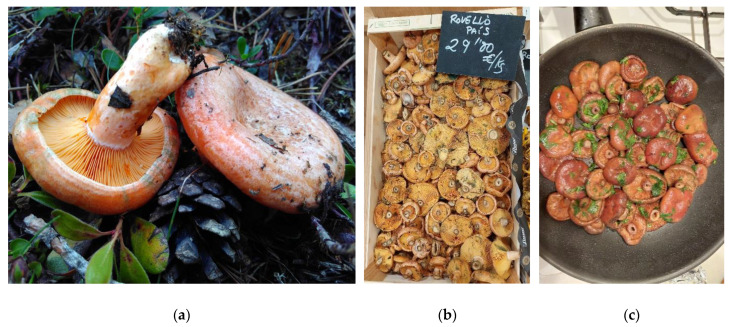
Wild edible fungi (WEF) of the *Lactarius* sect. *Deliciosi* ethnotaxon, which includes two of the main macromycetes cited in the Catalan linguistic area (CLA), *Lactarius sanguifluus* and *Lactarius deliciosus*: (**a**) *Lactarius deliciosus* in the forest. (**b**) “*Rovellons*” sold at a market in Vilafant (Alt Empordà), harvested in Catalonia. (**c**) Cooking “*rovellons*” traditionally with “*all i julivert*” [garlic (*Allium sativum*) and parsley (*Petroselinum crispum*)].

The most frequently cited species in the bibliographic corpus was *Lactarius sanguifluus* (Paulet) Fr., followed by *Macrolepiota procera* (Scop.) Singer and *Amanita caesarea* (Scop.) Pers. The latter shares third place with *Marasmius oreades* (Bolton) Fr., and *Tricholoma terreum* (Schaeff.) P. Kumm. and *Lactarius deliciosus* (L.) Gray are included among the WEF with more than 100 BR. Table 1 presents the WEF cited with 50 or more BR concerning edible mushrooms in the CLA, and their vernacular names; and Figure 4 and Figure 5 include pictures of the most cited WEF.

The taxonomic analysis of the documented WEF reveals that 87.62% of the cited taxa belong to the division Basidiomycota, whereas only 12.38% correspond to Ascomycota. These findings are consistent with other ethnomycological works (e.g., Ríos-García et al. [17]) and are explained by the relative visibility and accessibility of the typical fruiting bodies produced by Basidiomycota. Accordingly, 94.58% of the recorded mushrooms are epigeous, i.e., they develop their fruiting bodies above ground, while only 5.42% are hypogeous, with a subterranean fruiting body. This pattern reflects an ethnographic rationale that aligns with observations in the domain of ethnobotany, where the most visible plant parts (e.g., flowers, leaves, or fruits) are also the most frequently recognized and traditionally utilized [172]. Hence, it is unsurprising that the most widely known and documented edible mushrooms are those that can be readily observed, identified, and collected in the wild without requiring specialized or complex techniques. An exception is constituted by the hypogeous Ascomycota termed, in Catalan, “*tòfona*” (truffle) in the area studied, e.g., those from the genera *Tuber* or *Terfezia*, which are highly appreciated and collected with the help of animals, such as dogs or pigs.

The EMI, calculated as indicated in the Material and Methods Section, gives a value of 15.31%, meaning that one out of six/seven macromycete taxa present in the territory considered are known and used for food purposes. We did not find values for this index, whether under the new name and meaning we propose, or under the name of ethnobotanicity index limited to macromycetes. More studies on ethnomycology, including the calculation of this index, should be encouraged in order to compare the results in different areas.

Both the absolute number of edible taxa and the EMI (see Section 2.6) are much higher for fungi than for plants in the area studied. In a meta-analytic work, Gras et al. [173] found 291 wild edible plant taxa, with an ethnobotanicity index of 6.62%, whereas 643 taxa have been identified, leading to an index of 15.31% for wild edible mushrooms. Even if some of the mushrooms have been very rarely quoted as edible, and those which are really eaten frequently and regularly are much less than the total cipher, this could also apply, at least to some extent, to plants. In two Asian areas (much smaller than the one here considered), in which data of both wild edible plants and fungi are compared, the number of plant taxa was much higher than that of fungal ones (185/17, China, [174]; 110/49, Laos, [175]). In any case, this confirms that the CLA is a relevant mycophilous geographical and cultural territory, and highlights the important role of fungi in the local diet—likely greater than commonly assumed—opening the possibility of incorporating more fungi into the food market.

To refer to the 643 edible taxa, we collected 1562 Catalan names corresponding to 483 of them. The number of vernacular names per taxon ranges from 1 to 32. Only 11.98% of the taxa are associated with ten or more Catalan names, while 24.27% have only a single name. For 25.19% of taxa, no vernacular name was recorded, despite their recognition as WEF. The index of myconymic diversity is 2.43. As for the EMI, we did not find reported values for this index, and neither of the phytomymic diversity index adapted to fungi. However, we can state that the value here reported is higher than the very most of phytonymic diversity indices calculated for plant names in many regions of the area studied [23], claiming an important cultural consideration of mushrooms in this territory.

This myconymic diversity, on the one hand, is caused by the big distribution area of many taxa, so that they are the object of a dialectological variability, with different names or variants. On the other hand, it indicates a deep traditional knowledge of many fungi, and means the frequent collection, the high culinary consideration and, to sum up, the high cultural value attributed by people to WEF. Nevertheless, one point leads us to a reflection on a difference between phytonymy and myconymy in ethnobiology. Out of the 643 WEF detected, 162 do not have any folk name in the CLA. This quarter (25.19%) of non-named edible mushrooms is not paralleled in edible plants [173], where all, or almost all, plants used as food have folk names, in concordance with the fact that one of the first activities people do with objects is naming them, and that the uses tend to be more affected by changes than the names, which are the last piece information to disappear in cases of cultural erosion [23]. As reported in the present paper, myconyms in the CLA are numerous and varied, and they even make up part of sayings, as phytonyms do, such as “*Per Sant Josep, la múrgola treu el bec*” (“For Saint Joseph, the morel sticks out its beak”, uniting the similarity of morel’s cap and a bird’s beak, and indicating spring as the season of this mushroom), collected in north-eastern Catalonia around 2000. This 25% gap makes us believe that those unnamed and potentially used WEF are rarer, concerning distribution and abundance, and more rarely eaten than those having one or many more folk names. In any case, this could be at least partly a matter of the under-representation of ethnomycological studies, at least in Europe, suggesting that new research is necessary in this field.

Not surprisingly, *Lactarius sanguifluus* (Paulet) Fr. is the taxon with a highest number of folk names, which is also the top one as per number of BR, indicating that is the most (or, at least, one of the most) well-known and appreciated in the area studied. Indeed, the “*rovellons*”, which include different species of the genus *Lactarius*, evoke strong interest also because of their commercial importance, since more than half of the WEF sold in the biggest market in Catalonia (Mercabarna) belong to this genus [176], of which *L. sanguifluus* is one of the principal edible species.

*Armillaria mellea* (Vahl) P. Kumm., with 30 Catalan names, and *Morchella esculenta* (L.) Pers. and *Hydnum repandum* L., with 29 each, followed by *L. deliciosus* (27 vernacular names) are the most myconym-rich taxa. *Morchella esculenta* (L.) Pers. taxa belong to the Ascomycota, reflecting the strong knowledge surrounding this fungal division. Although it is less common compared to the Basidiomycota—which includes most of the classic edible macromycetes—it is still well-recognized and valued.

### 3.3. Nutritional Values of WEF from the CLA

From the 169 articles selected in the second search, nutritional information has been obtained from 228 taxa of edible fungi present in the CLA. Based on the 643 taxa compiled in the final corpus of WEF, scarcely more than one third (35.46%) of the taxa have nutritional data information. This percentage shows a clear lack of systematic nutritional studies for many species of mushrooms in this area and may limit their recognition as valuable food resources within dietary guides or food composition tables. Although this proportion may seem low, it can still be considered relevant in terms of its implications for public health and market potential. We encourage further nutritional composition studies in taxa lacking such information. The objective of the present paper was to describe the current knowledge of nutritional data concerning WEM, with the purpose of fostering future research in this applied field.

As Kalač [177] pointed out, nutritional data from mushrooms should be taken as approximate values for some reasons, such as the fact that the mushroom’s chemical variability is greater than in plants, since each macromycete can result from the cross-breeding of different hyphae (with distinct genotypes).

The articles that reported most nutritional values of the WEF in the CLA were Vetter [77], who reported the P content of 37 taxa, followed by Alonso et al. [54], Kaya et al. [97] and Piepponen et al. [85], who reported 27 taxa each. Another 28 articles reported 10 or more taxa, whereas 3 taxa per article is the median.

Most of the reported nutritional values (80.18%) refer to the entire fruiting body of the macromycete. In only 6.22% of the cases, the analysis focused solely on the cap, while 5.91% of the studies examined the stipe. Alonso et al. [54], Arvay et al. [80], and Melgar et al. [69] chose to analyse the nutritional values of the hymenophore and the rest of the fruiting body separately. In greater detail, 80.41% of the samples were dried before analysing, 7.53% were processed, 7.07% were frozen, and 2.15% were analysed raw. Only in 2.84% of the cases could we not establish the processing method of the sample.

**Figure 6:**
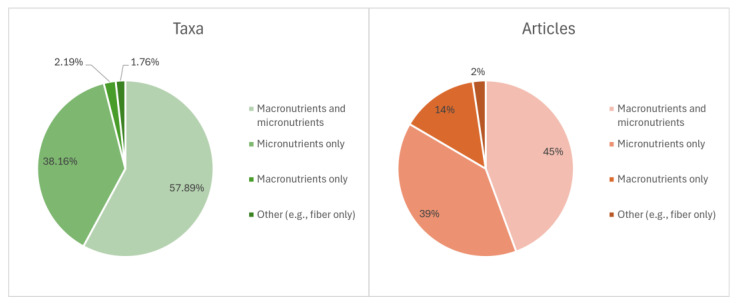
Visual representation of the percentage of taxa (**left graph**) and articles (**right graph**) reporting nutritional data on macronutrients, micronutrients or both, based on the compiled literature.

A total of 132 WEF from the CLA contained macro- and micronutrient information. The distribution of taxa and articles reporting macronutrient, micronutrient or both types of data is represented in Figure 6. However, among the most cited in the CLA, *Chroogomphus rutilus* (Schaeff.) O.K. Mill. (70 BR) and *Lyophyllum decastes* (Fr.) Singer (53 BR) only reported micronutrient information, such as 85 more WEF. *Suillus collinitus* (Fr.) Kuntze, *Paralepista flaccida* (Sowerby) Vizzini and *Suillus mediterraneensis* (Jacquet. & J. Blum) Redeuilh, three WEF from the CLA, with 27, 22 and 12 BR, respectively, only reported macronutrient information, such as two more WEF. As mentioned, Table 1 offers the articles containing nutritional values of the WEF from the CLA with 50 or more BR, while the detailed information is available in the “Data Availability Statement”.

It should be noted that *Hygrophorus latitabundus* Britzelm. and *Hypomyces lateritius* (Fr.) Tul. and C. Tul. were the only two taxa with 50 or more BR without nutritional values reported, making them perfect candidates for future research.

This information gap makes them excellent candidates for future analytical studies, both to cover a lack of knowledge and to value native species that have not yet been sufficiently studied from a nutritional point of view.

A particular case of highly cited WEF in the CLA is *Gyromitra esculenta* Pers. ex Fr. (26 BR), which only offers a fibre value, not nutritional ones. This WEF is considered controversial, due to reports of its toxicity, yet it continues to be widely consumed, often linked to cultural and traditional practices. It is important to carry out an in-depth analysis of this taxon, including an evaluation of its variability before and after processing. Traditional preparation methods—such as drying and boiling before cooking, boiling only, or drying and cooking directly—should be considered, along with modern practices like freezing, with or without prior boiling.

Also linked to the CLA, the countries that reported more nutritional values from WEF between 1979 and 2025, were Türkiye, with 131 taxa, followed by India with 53 taxa, Portugal with 48 taxa, Hungary with 45 taxa, and Spain with 44. It is not surprising that the country that has contributed the most on reporting nutritional data is located in the Mediterranean area, and that in third position is a territory geographically and culturally close to the CLA, which, like these, has at least some areas with a strong mycophile tradition.

Focusing on the nutritional values reported, information about micronutrients was higher than for macronutrients; see Table 2 for specific information on food components and BR. We also reported some information related to trace elements, contaminants and other potentially toxic elements, due to macromycetes’ characteristics. We consider it important to collect this data since mushrooms are provided with efficacious mechanisms to accumulate these elements from soil [150].

The taxa with the highest energy presence, in terms of calories, is *Macrolepiota mastoidea* (Fr.) Singer with 700.96 kcal/100 g DM, considering that the mushroom was processed (cooked); without any processing method, dried *Tirmania pinoyi* (Maire) Malençon apported 651.50 kcal/100 g DM. Energy was reported as the original source, in kJ or kcal (1 kcal = 4.2 kJ), since it is preferable not to calculate those values using the energy conversion factors [44]. Relating to calories, fats are the elements that provide more calories per gram (9 kcal/g) [178], so it is not surprising that one of the highest values of fat is the processed *Macrolepiota mastoidea* (Fr.) Singer mentioned before, with 63.03 g/10 g DM. However, the highest value was for dried *Hericium erinaceus* (Bull.) Pers., with 89.70 g/100 g DM. From 331 BR for fat, in 242 BR we were able to calculate the g/100 g of edible portion (since moisture data was also reported), and 90.50% of these macromycetes reported less than 3 g of fat per 100 g of edible portion, meaning that the claim “low fat” can be applied to the majority of WEF [179].

Macromycetes are characterized by being rich in protein, and some authors reported a range of protein between 20 and 25 g/100 g DM. However, it is also considered that in some studies, the nutritional value can be overestimated due to the application of nitrogen to protein conversion factor, considered to be 4.38 for macromycetes or 4.16 for WEF [44,177]. The data obtained presents more than 25 g/100 g DM of protein in 53 WEF, with dried *Sparassis crispa* (Wulfen) Fr. Being the taxon with the highest value (83.40 g/100 g DM).

In macromycetes, glycogen is the main energy reserve, and their carbohydrate composition –monosaccharides, oligosaccharides, and polysaccharides– differs from that of plants, which typically store energy as starch [180]. We collected the data as total carbohydrates: *Neoboletus erythropus* (Pers.) C. Hahn was the taxon with a highest value and *Lactarius semisanguifluus* R. Heim & Leclair the lowest. A more thorough investigation into the distinctions between sugars and polysaccharides, together with the influence of processing techniques, should be undertaken to support further conclusions.

As non-digestible carbohydrates, we can comment on fibre, which is a mix of non-starch polysaccharides (NSPs), fructo-oligosaccharides (FOSs), galacto-oligosaccharides (GOSs) and other resistant oligosaccharides (known as resistant starch) [181]. Wild edible fungi (WEF) are rich in fibre, and principally in insoluble fibre, which is considered to contribute to a good health condition of the colon, since it softens stools, increases stool bulks, and reduces the postprandial blood glucose by increasing the viscosity of foods and gastric contents [180]. However, as seen in Table 1, fibre was not among the most reported values in WEF, and further research may be interesting, due to the health benefits of this food component.

Macromycetes usually contain 5–12 g/100 g DM of ash; however, contents of more than 20 g/100 g DM were reported in the literature for several WMs (wild mushrooms), as Kalač pointed out [177]. Variability reported in data from WEF range between 0.18 and 38.90 g/100 g DM, providing information about minerals such as *Clavulina cinerea* (Bull.) J. Schröt. containing the major Ca content; *Fistulina hepatica* (Schaeff.) was reported with major contents of K; and *Volvopluteus gloiocephalus* (DC.) Vizzini contained major contents of Mg, Na, and P. Data was recorded for other minerals and trace elements, since mushrooms are a good source due to their capacity to accumulate these elements in higher concentrations than agricultural crops, vegetables, fruit or animals [182]. This characteristic may result in the bioconcentration of contaminants such as As, Cd or Pb, as well as potentially toxic elements like Al and Ni, with levels influenced by environmental factors [182]. Even though some values for these food elements have been reported, some toxic metals like Cd, Hg and Pb can be leached away through culinary processes [183]. Short-term boiling is more effective than soaking at room temperature, and frozen mushroom slices—due to tissue damage—release a higher proportion of these elements compared to fresh, dried, or intact mushrooms [183]. These methods are part of traditional culinary practices, as mushrooms are rarely consumed raw in the CLA. Preservation techniques like air-drying—typically followed by soaking prior to cooking—or freezing, are widely used. Although some flavour compounds are also lost through leaching [183], WEF continue to be used and consumed following these practices—not only to enhance the taste and aroma of dishes, but also, whether consciously or not, to help remove certain toxic substances.

Regarding vitamins and provitamins, 275 BR were recorded, principally from ascorbic acid (vitamin C) and total tocopherols. Some articles reported tocopherol values in the different forms; however, data were only collected as total tocopherols, being the sum of α- to δ-tocopherols and α- to δ-tocotrienols [177]. However, α-tocopherol is considered the active form of vitamin E, and other equivalents should not be used [44]. WEF with highest values of tocopherols were *Craterellus cornucopioides* (L.) Pers., *Imleria badia* (Fr.) Vizzini, and *Boletus edulis* Bull, which also recorded the highest value of vitamin C. All WEF that recorded ascorbic acid levels were raw, dried or frozen, except for *Boletus edulis* Bull. and *Imleria badia* (Fr.) Vizzini, which reported vitamin C levels after blanched. Even cooking treatments and usual preservation methods decrease levels of this vitamin—as those of other water-soluble vitamins—[177], the amount of L-dehydro-ascorbic acid increases during cooking or processing [44], which may explain the values recorded in processed WEF.

Among the group B vitamins (thiamine, riboflavin, niacin, vitamin B_6_, and vitamin B_12_) recorded, it is important to emphasize vitamin B_12_, or cobalamin, which is not present in plants. Palazzolo et al. [71] analysed the values of B_12_ of five WEF, which reported a range of 0.89–1.7 µg/100 g of edible portion; and *Macrolepiota procera* (Scop.) Singer contained the highest amount. In adults, the adequate intake (AI) for vitamin B_12_ is 4 µg/day [184], meaning that values reported will cover between 22 and 43% of AI, considered an interesting choice for vegan or vegetarian diets. However, this data was analysed in raw mushrooms, and only in one source, since is not enough to consider WEF as a source of vitamin B_12_, and further studies should be conducted regarding this food component and vitamin B-group vitamins in general.

β-carotene is the most potent precursor of vitamin A; however, its bioavailability in humans is not determined [177]. *Lactarius deliciosus* (L.) Gray and *Lactifluus piperatus* (L.) Roussel reported the highest values of this provitamin.

Finally, macromycetes can provide two very interesting provitamins: ergosterol and cholecalciferol, which convert to vitamin D_2_ and D_3_, respectively. Three articles [50,137,139] reported ergosterol data, and two [71,143] cholecalciferol, with *Suillus bellinii* (Inzenga) Kuntze and *Craterellus cornucopioides* (L.) Pers. Being the two WEF that reported the highest values, respectively. Ergosterol plays a similar function in fungi, like cholesterol in animals, strengthening cell walls, modulating membrane fluidity, and assisting intercellular transport [185]. In animals, vitamin D_3_ is the most common form, while in macromycetes it is the D_2_ form, but they also contain D_3_ and D_4_. Vitamin D_2_ content may diminish with storage and cooking [185], and macromycetes produce vitamin D_2_ if they are exposed to UV light during a certain time. In adults, the AI for vitamin D is 15 µg/day [186]. Values reported for five WEF [66] ranged from 9.50to 8.90 µg/100 g per edible portion, covering from 59.99% to 63.33% of AI. Since bioavailability of vitamin D_2_ from macromycetes has been demonstrated in rats and humans [185], further studies must provide information about WEF vitamin D as a non-animal source.

## 4. Conclusions

This work shows that the CLA is an importantly mycophile region, with a high number of macromycetes used for food purposes, traditionally named with a high number of myconyms, and with high ethnomycological indices, indicators of the relevance of mushrooms in the relationship between people and biodiversity in the area studied.

Ethnomycological research is much less practised than ethnobotanical research worldwide, and particularly in Europe, where this kind of investigations should be encouraged.

In our extensive review, a significant corpus of nutritional data on WEF has been compiled, particularly concerning micronutrients. Among all of these, WEF are good sources of non-plant vitamins, like vitamin D and vitamin B_12_, which provide a richer and complete diet, diminish meat consumption, and link to the so-called Mediterranean diet.

Nevertheless, nutritional data on fungi are still scarce and, additionally, disperse and difficult to standardise. The Food and Agriculture Organisation (FAO) of the United Nations does not mention fungi at all in its biodiversity database (BioFoodComp4.0). Whereas a first comprehensive attempt has been made to establish the nutritional composition of wild edible plants in the Mediterranean Region [187], no parallel works on WEF are available. It is urgent that the nutritional value of mushrooms be assessed, since mushroom consumption is elevated, although heterogeneously distributed, worldwide. Consumption per capita is around 30–40 g/person/year, and in the US and many European countries, around 3 kg/person/year (a value considered very low compared to the 10 kg/person/year in China) [188]. Finally, recent projects like the Periodic Table of Food Initiative (PTFI) [189], may be a good opportunity to include WEF, since some cultivated mushrooms have already been analysed.

## Figures and Tables

**Table 1:** Wild edible-fungal taxa from the CLA with 50 or more bibliographic reports (BR), vernacular names, macronutrients or micronutrients reported, number of articles with nutritional values (Num. Art.), and references. Detailed nutritional information of macronutrients and micronutrients for all the taxa can be consulted in CORA (see Data Availability Statement Section).

Taxa	BR	Vernacular Names (In Catalan, Unless Another Language Is Indicated)	Macro-Nutrients	Micro-Nutrients	Num. Art.	References
*Lactarius sanguifluus* (Paulet) Fr.	115	Bolet de sang, esclata-sang, esclata-sang blanc, esclata-sang de migjornet, esclata-sang de murta, esclata-sang de pi, esclata-sang de sivina, esclata-sang de xipell, esclata-sang d’hivern, esclata-sang mascle, esclata-sang murta, esclata-sang sanguinós, esteper, gallufer, lleterola, mare del rovelló, pebràs, pebre, pinatell, putifler, rovelló, rovelló blanc, rovelló de llistó, rovelló de plana, rovelló de sang, rovelló de solell, rovelló d’espígol, rovelló esclata-sang, rovelló vinader, rovelló vinagrer, *seta* (Spanish), vinader	🞬	🞬	7	[47,48,49,50,51,52,53]
*Macrolepiota procera* (Scop.) Singer	110	Apagallums, bolet de frare, calceta, cama-sec, cama-seca, capell de senyor, cogoma, cogombre, cogomella, cogomella vera, cohoma, coloma, farinosa, maneta, massa, *mazza di tamburo* (Italian), paloma, pamperol, pampinella, paraigua, para-sol, pimpinella, pimpinella farinosa, pota d’ase, senyal d’alzina, *sombrilla* (Spanish)	🞬	🞬	33	[49,54,55,56,57,58,59,60,61,62,63,64,65,66,67,68,69,70,71,72,73,74,75,76,77,78,79,80,81,82,83,84,85]
*Amanita caesarea* (Scop.) Pers.	108	Bolet d’or, cocou, monjola, oriol, oronja, ou de monjola, ou de rei, ou de reig, quicou, rei, reig, rovell d’ou	🞬	🞬	4	[72,86,87,88]
*Marasmius oreades* (Bolton) Fr.	108	Cama-sec, cama-sec de prat, caramanyola, carmanyola, carrereta, carrerola, carretera, correjola, correola (spanish), correrola, corretjola, corriola, corrioleta, esclata-sang caminer, fals moixerdó, fals moixernó, moixeriga, moixernó, moixina, paraigüet	🞬	🞬	10	[54,66,76,79,80,85,89,90,91,92]
*Tricholoma terreum* (Schaeff.) P. Kumm.	107	Bolet de rosada, bolet negre, brunet, bruneta, esteperol, fraret, fredeluc, fredolí, fredolic, fredolic mascle, fredolís, fredoluc, gírgola d’estepa, gírgola d’estèpera, griset, moreneta, morro d’ovella, negrentí, negret, negric, *negrito* (Spanish), orella d’estèpera, palometa	🞬	🞬	10	[75,77,83,92,93,94,95,96,97,98]
*Lactarius deliciosus* (L.) Gray	102	Bolet comestible, esclata-sang, esclata-sang de bruc, esclata-sang de bruc femella, esclata-sang foraster, esclata-sang vermell, esteper, paratge, peratxe, pinatell, pinenc, pinenca, pinetell, pinetenc, pinetenca, rodelló, rovelló, rovelló de boixera, rovelló de bruc, rovelló de llicorella, rovelló de monte, rovelló d’obaga, rovelló d’ombria, rovelló pinetell, rovellola, vermell	🞬	🞬	30	[47,48,49,50,51,52,53,54,57,69,70,78,79,80,81,83,85,92,95,96,97,99,100,101,102,103,104,105,106,107]
*Morchella esculenta* (L.) Pers.	94	Aligany, aragall, arigany, brújola, greixot, marúgola, mírgola rossa, morga, morúgola, murga, murga de campana, murga de cap rodó, múrgara, múrgola, múrgola cònica, múrgola de cap rodó, múrgola de rec, múrgola de ribera, múrgola fosca, múrgola grisa, múrgola rossa, murguela grisa, murguela rossa, múrmola, múrmola blanca, múrmola de freixe, múrmola rossa, rabassola, vírgula	🞬	🞬	12	[49,52,66,76,83,96,108,109,110,111,112,113]
*Hygrophorus russula* (Schaeff. ex Fr.) Bataille	93	Bolet d’alzina, cabrit roig, carlet, carlet de mura, carlet vermell, carlí, carlista, carló, carlot, cruelda, escarlet, escarlet ver, escarlet vermell, escarlot, escarlot vermell, llenega vermella, rovelló d’alzina, vermella, vinassa, vinós, vinosa	🞬	🞬	2	[87,114]
*Cantharellus cibarius* Fr.	89	Agerola, cama-seca, cama-seca de mata, carn de gallina, cresta de gall, espicatornell, gerola, ginesterola, girola, *girolle* (French), picanell, picornell, picornell d’ullastre, picornell foraster, rossinyol, rossinyol comú, torrendó, torrentó, vaqueta	🞬	🞬	32	[52,54,58,66,72,74,76,79,80,81,85,86,87,88,91,103,115,116,117,118,119,120,121,122,123,124,125,126,127,128,129,130]
*Craterellus lutescens* (Fr.) Fr.	87	Cama tarongina, camagroc, camagroga, cama-roja de bruc, cama-seca de bruc, cama-seca d’hivern, ginesterola de pi, indi, misto, moixernó de bosc, peu de rei, picornell de càrritx, picornell de pi, pota de perdiu, rossinyol de pi, rossinyolic, rossinyolic camagroc, sip, tarongina, trompeta, vaqueta de pi	🞬	🞬	1	[103]
*Hydnum repandum* L.	84	Agulla, agulles, agulleta, agulletes, blanquet, boïna, boixet, bolet de prat, buina, dent de rata, llémena, llengo de bou, llengua boïna, llengua bovina, llengua de bou, llengua de vaca, llengua de vedella, llengüeta, peu de cabrit, peu tort, picaronell, picarronell, picornell, picornell blanc, picornell pelut, pied-de-*mouton* (French), punxeguda, vaquet, vaqueta	🞬	🞬	15	[52,54,55,69,72,85,92,98,102,103,118,123,125,130,131]
*Armillaria mellea* (Vahl) P. Kumm.	82	Alzinó, alzinoi, aulinell, bolet d’ametler, bolet de roure, bolet de soca, bolet de soca d’alzina, bolet de soca d’aulina, cama-sec de soca, cama-seca de soca, flora d’arbre, flota d’alzina, flota d’arbre, flota de noguera, flota de pollancre, flota de roure, flotona, gírbola d’alzina, gírbola d’aulina, gírbola de roure, gírgola d’alzina, mata de roure, olinell, pollancó, pollancró, pollarenca, polletó, rourenc, rouró, socada d’alzina	🞬	-	20	[55,59,77,78,79,81,85,87,91,97,98,114,118,125,126,132,133,134,135,136]
*Pleurotus ostreatus* (Jacq.) P. Kumm.	79	Auriana, bolet de ventall, clopí, clopissa, clopissó, flora d’orella, flota d’arbre, giragola, gírbola, gírbola d’arbre, gírgola, gírgola d’arbre, gírgola de beç, gírgola de poll, gírgola de pollancre, orella, orellana, orellana comuna, orellana d’arbre, orellana de poll, orellana de polla, orellana de pollancre, orelleta, oriana de polla, pollancró, vimequer	🞬	🞬	22	[53,54,58,66,76,77,78,83,84,90,93,96,97,98,109,120,133,137,138,139,140,141]
*Craterellus cornucopioides* (L.) Pers.	73	Alzinoia, corn, corn de l’abundància, corneta, orella d’ase, orella de burro, orella de ruc, rossinyol negra, rossinyol negre, trompeta, trompeta de la mort, trompeta de perdiu, trompeta dels morts, trompeta negra, ull de perdiu, vaqueta negra	🞬	🞬	13	[58,67,72,78,79,85,91,103,118,119,130,142,143]
*Chroogomphus rutilus* (Schaeff.) O.K. Mill.	70	Bec de perdiu, bitxac, cabridet, cama de perdiu, cama-roja, *carnero* (Spanish), fetge, fetget, pebrasset de moro, peu de perdiu, pota de perdiu, ull de perdiu	-	🞬	4	[72,90,97,144]
*Boletus edulis* Bull.	69	Bolet de bou, bolet de *porcino* (Italian), bolet porcí, cep, cep de bordeus, cepa, *cèpe* (French), ceperó, cigró, corball blanc, *porcino* (Italian), siureny, siureny de baga, siurenya, siuró, siurol, surenc, sureny, sureny de baga, trompellot	🞬	🞬	47	[52,54,55,58,59,65,68,69,70,73,75,77,79,80,81,84,85,86,105,109,119,120,123,124,125,126,127,128,129,131,145,146,147,148,149,150,151,152,153,154,155,156,157,158,159,160,161]
*Agaricus campestris* L.	61	Bola de neu, bolet, bolet blanc, bolet blanc matós, bolet blanc salvatge, bolet de camí, bolet de porc, camperol, camperol comú, comaga, culblanc, fumat, gírgola blanca, pamperol, pexigà, rovellol, rubiol, rubirol, terrerol, terronenc, xampinyó, xampinyó de bosc, xampinyó silvestre	🞬	🞬	15	[49,54,58,59,66,67,69,73,76,79,88,90,112,133,162]
*Boletus aereus* Bull.	61	Cap de negre, cep, cep negre, cigró, mollerol, padrina, pixacà, siurac, siureny, siureny fosc, siuró, siurol, surenc, sureny, sureny fosc	🞬	🞬	8	[54,87,119,145,148,151,156,158]
*Calocybe gambosa* (Fr.) Donk	60	Bolet de Sant Jordi, cama-seca blanca, moixern de primavera, moixernó, moixernó de prat, moixernó de primavera, moixernó de Sant Jordi, moixernó ver, moixeró, moixeró blanc, moixeró de primavera, moixerró, moscardó, *perretxico* (Basque)	🞬	🞬	5	[58,97,128,136,163]
*Infundibulicybe geotropa* (Bull.) Harmaja	59	Barretet de frare, campana, candela, candela d’arboç, candela de bruc, caperons, frare, gírgola de bruc, moixernó de candela, moixernó de Sant Miquel, moixernó de tardor, moixeró de Sant Miquel, moixeró de tardor, orella de frare, orellana de bruc, pampa, porrosa, tassa de bruc	🞬	🞬	3	[71,89,132]
*Hygrophorus latitabundus* Britzelm.	58	Bateó, bavallosa, bavós, bavós rabassut, bavosa, cabrit, caramellosa, gírgola de llim, gírgola de mata, gomera, llenega, llenega negra, llenegall, mocosa, mocosa grisa, mocosa negra, pegalosa, senyoreta	-	-	0	-
*Suillus granulatus* (L.) Roussel	57	Bolet de bou, bolet de pi, bolet de vaca, cabreta, esponja, groguet, mataparent, moixí, moixí granellut, moixina, molleric, molleric granellut, molleric ver, mollic, pebrada, pinetell, pixacà, vaca, vacassa	🞬	🞬	12	[62,66,71,75,76,77,83,84,92,128,164,165]
*Hypomyces lateritius* (Fr.) Tul. and C. Tul.	56	Esclata-sang de tot l’any, esclata-sang mascle, esclata-sang putifler, mare de la pinenca, mare de rovelló, mare del pinetell, mare del rovelló, pinenca tofonera, pinetell boixat, pinetella, rovelló mascle, rovellola, rovellona, rovellonera, tofana	-	-	0	-
*Russula cyanoxantha* (Schaeff.) Fr.	55	Blava, blavet, blaveta, camadolça, cualbra, cualbra blava, cualbra llora, cualbra morada, cualgra llora, llaurell, llora, llora aspra, llora blava, llora carbonera, llora verda de peltereau, llorell, palomí, puagra, puagra llora, raurell, terradolça, terrandolça	🞬	🞬	7	[54,69,73,77,98,119,166]
*Tricholoma equestre* (L.) P. Kumm.	54	Camagroc, canari, carbonera groga, carboneta groga, carlet groc, escarlet groc, fredolic, fredolic groc, groguet, groguí, groguillo, negret, pixaconill, rentiscle, rosteta, taverniscle, verderol, xamardiscle, xavarnol, xaverniscle, xaverníscola	🞬	🞬	4	[49,54,102,167]
*Lyophyllum decastes* (Fr.) Singer	53	Agret, bolet de bruc, bolet de pila, carner, flota carnera, flota de bruc, flota de bruc cendrosa, flota de mòdega, flota d’olina, flotona de bruc, flotona de carbonera, gírbola de bruc, gírgola de bruc, gírgola de mòdega, gírgola d’estepa, moixernó d’estepa, pom de terra, soca de bruc, soca de carnera	-	🞬	3	[130,141,144]
*Suillus luteus* (L.) Roussel	52	Bolet de bou, bolet de vaca, mataparent anellat, moix, moixí, moixí de calceta, molleric, molleric calçat, molleric de calceta, mollic, pinatell de calçeta, pinetell, pinetell de calceta, vaca	🞬	🞬	15	[49,66,76,81,83,85,90,95,96,97,104,106,129,133,168]
*Lycoperdon perlatum* Pers.	51	Bufa, bufa de bou, bufa de jai, bufa de jaia, bufa de monja, bufa del dimoni, esclatabufa, fum de terra, fumosa, llufa de llop, pet de bou, pet de ca, pet de frare, pet de llop, pet de llop perlat, pet de monja, pet de moro, pet de vella, pet del diable	🞬	🞬	11	[61,66,76,78,79,81,91,116,118,135,139]
*Coprinus comatus* (O.F. Müll.) Pers.	50	Aglà d’alzina, bolet de femer, bolet de fems, bolet de merda, bolet de tinta, bolet negre, coprí menut, coprí pelut, paraigua, pixacà barbut	🞬	🞬	13	[49,54,61,69,85,90,92,97,133,136,141,169,170]
*Russula delica* Fr.	50	Blanqueta, blava, bolet blanc, bolet fort, *campanilla* (Spanish), cogoma, cualbra blanca, esclata-sang blanc, esclata-sang d’alzina, forta, llora blanca, pebràs, pebràs comú, pebràs ver, pebrassa blanca, pebrotasso, terlandòs, terrandòs	🞬	🞬	11	[47,50,66,76,89,97,98,125,144,147,171]

🞬: data available (macro- and/or micronutrient values reported in the literature); -: no data available (no values reported in the literature).

**Table 2:** Food components and their bibliographic reports (BR).

Food Components	Units	BR	Range
Energy kJ	kJ (original)/100 g DM ^1^	52	74.07–1736.59
Energy kcal	kcal (original)/100 g DM	194	20.38–700.96
Water/Moisture	g/100 g FM ^2^	306	2.17–96.60
Protein: total	g/100 g DM	423	1.21–83.40
Fat: total	g/100 g DM	331	0.09–89.70
Carbohydrates: available	g/100 g DM	338	0.85–89.80
Fibre: total dietary	g/100 g DM	98	0.28–83
Insoluble fibre	g/100 g DM	57	3.92–50.60
Soluble fibre	g/100 g DM	23	0.75–24
Ash	g/100 g DM	366	0.01–38.90
Ca	mg/100 g DM	518	0–19,100
Cu	mg/100 g DM	753	0–340
Fe	mg/100 g DM	682	0–15,100
K	mg/100 g DM	489	0.25–32,789.17
Mg	mg/100 g DM	528	0.01–9180
Mn	mg/100 g DM	619	0–315.07
Na	mg/100 g DM	460	0–16,100
P	mg/100 g DM	352	0.03–782,000
Se	mg/100 g DM	247	0–450
Zn	mg/100 g DM	772	0–740
Al	µg/100 g DM	232	160–1,510,000
As	µg/100 g DM	134	0–280
Cd	µg/100 g DM	501	0.31–6,128,000
Co	µg/100 g DM	251	0.40–1,180,000
Cr	µg/100 g DM	356	0.16–20,300
Ni	µg/100 g DM	367	1–17,415
Pb	µg/100 g DM	456	2–1,050,000
Thiamine	mg/100 g DM	16	0.14–17.03
Riboflavin	mg/100 g DM	13	0.06–4.97
Niacin	mg/100 g DM	17	0.66–77.64
Vitamin B_6_	mg/100 g DM	8	0–1.13
Vitamin B_12_	µg/100 g DM	5	3.59–23.84
Ascorbic acid (vitamin C)	mg/100 g DM	76	0.33–5320
Folate	µg/100 g DM	2	87.10–159
Vitamin A	µg/100 g DM	6	0–38,360
β-carotene	µg/100 g DM	36	0.27–4835
Vitamin D_2_ (ergosterol)	µg/100 g DM	15	740–240,900
Vitamin D_3_ (cholecalciferol)	µg/100 g DM	6	2.07–1520
Vitamin E (as total tocopherols)	µg/100 g DM	75	0–47,900

^1^ DM = Dry matter; ^2^ FM = Fluid matter.

## Data Availability

All data are available in CORA (the Catalan Open Research Area). Vernacular names of WEF in CLA at https://doi.org/10.34810/data2422 and Nutritional values of WEF in CLA at https://doi.org/10.34810/data2423.

## Abbreviations

The following abbreviations are used in this manuscript:

AI	Adequate intake
BC	Biblioteca de Catalunya
BR	Bibliographic Report
CLA	Catalan Linguistic Area
CORA	Catalan Open Research Area
CRAI	Centre de Recursos per a l’Aprenentatge i la Investigació
DM	Dry Matter
EI	Ethnobotanicity Index
EMI	Ethnomycoticity Index
FM	Fluid Matter
UB	Universitat de Barcelona
WEF	Wild Edible Fungi
